# Integrating landscape ecology into generic surveillance plans for bark‐ and wood‐boring beetles

**DOI:** 10.1002/eap.70194

**Published:** 2026-03-09

**Authors:** Davide Nardi, Davide Rassati, Andrea Battisti, Manuela Branco, Claudine Courtin, Massimo Faccoli, Nina Feddern, Joseph A. Francese, Emily Franzen, André Garcia, Filippo Giannone, Martin M. Gossner, Mats Jonsell, Chantelle Kostanowicz, Matteo Marchioro, Petr Martinek, Ann M. Ray, Alain Roques, Jon Sweeney, Kate Van Rooyen, Vincent Webster, Lorenzo Marini

**Affiliations:** ^1^ Department of Agronomy, Food, Natural Resources, Animals and Environment (DAFNAE) University of Padova Legnaro Padua Italy; ^2^ Centro de Estudos Florestais, Laboratório Associado TERRA, Instituto Superior de Agronomia Universidade de Lisboa Lisbon Portugal; ^3^ Institut National de Recherche pour l'Agriculture, l'Alimentation et l'Environnement (INRAE), UR 0633 Zoologie Forestière Orléans France; ^4^ Forest Entomology Swiss Federal Institute for Forest, Snow and Landscape Research WSL Birmensdorf Switzerland; ^5^ USDA‐APHIS‐PPQ‐S&T Forest Pest Methods Laboratory Buzzards Bay Massachusetts USA; ^6^ USDA APHIS PPQ S&T Bethel Field Station Bethel Ohio USA; ^7^ Department of Biology Xavier University Cincinnati Ohio USA; ^8^ Independent Researcher Padova Italy; ^9^ Department of Environmental Systems Science Institute of Terrestrial Ecosystems, ETH Zürich Zürich Switzerland; ^10^ Department of Ecology, SLU Uppsala Sweden; ^11^ Natural Resources Canada, Canadian Forest Service, Atlantic Forestry Centre Fredericton New Brunswick Canada; ^12^ Department of Forest Protection and Wildlife Management, Faculty of Forestry and Wood Technology Mendel University in Brno Brno Czech Republic

**Keywords:** alien species, biosecurity, Coleoptera, generic surveillance, landscape ecology, sample coverage, sampling effort, woodborers

## Abstract

International trade poses a growing threat to global biosecurity, with bark‐ and wood‐boring beetles representing a major concern for forest health. Non‐native species are frequently introduced at points of entry, where populations can establish in the surrounding landscape. To improve early detection, generic surveillance programs use traps in these high‐risk areas, collecting a broad spectrum of species. These traps also capture native beetles, providing insights into the potential species pool that could become exotic elsewhere. However, implementing effective landscape‐wide surveillance within reasonable resource limits remains challenging. In this study, we used trapping data of Cerambycidae and Scolytinae from 11 high‐risk areas across Europe and North America to develop practical recommendations for generic surveillance at multiple spatial scales. Specifically, we attempted to address two key questions: (1) how to maximize the single‐trap efficacy depending on the trap surroundings; and (2) how many traps should be used in a landscape‐wide sampling depending on landscape composition. Under budget constraints, we recommend prioritizing trap placement within forest patches and avoiding locations surrounded by roads or buildings. Urban‐dominated landscapes required greater sampling effort (i.e., more traps) than forest‐dominated landscapes. Deploying fewer than four traps per square kilometer might lead to an incomplete representation of the local bark‐ and wood‐boring beetle community, losing about 30%–50% of species. Overall, our findings highlight the importance of incorporating landscape ecology into generic surveillance planning to optimize trap effectiveness within resource limitations.

## INTRODUCTION

Insect invasions threaten agricultural and forestry sectors worldwide (Bradshaw et al., 2016; Brockerhoff & Liebhold, 2017; Pyšek et al., 2020). The constant increase in global trade over the last hundred years has facilitated the movement among continents and the establishment of an increasing number of exotic insects (Seebens et al., 2017; Pureswaran et al., 2022; Fenn‐Moltu et al., 2023; Nardi et al., 2025). Coleoptera, especially bark‐ and wood‐boring beetles (BWBB), have expanded their global distribution because of these processes (Eyre & Haack, 2017; Lantschner et al., 2020). Hidden in live plants or wood‐packaging materials (Liebhold et al., 2012; Meurisse et al., 2019), they are commonly introduced at entry points (Brockerhoff, Bain, et al., 2006; Haack, 2006; Wu et al., 2017), often circumventing the visual inspections routinely applied on imported commodities. As these invasions can strongly impact the local economy, affect ecosystem services, and even disrupt entire forest ecosystems (Lovett et al., 2016; Ramsfield et al., 2016), biosecurity plays a key role in reducing the risk of new introductions and managing established invasive species (Nahrung et al., 2023).

Biosecurity is a continuum of pre‐border, border, and post‐border measures (Arndt et al., 2024; Hulme, 2014; Hulme et al., 2023). National plant protection organizations largely rely on post‐border measures targeting high‐risk areas and their surroundings to detect non‐native BWBB species (Dodds et al., 2024; Fiala & Holuša, 2023; Rabaglia et al., 2019). Among the various tools and approaches developed over the years (Larson et al., 2020; Poland & Rassati, 2019), generic surveillance based on traps baited with multi‐lure blends is becoming increasingly important worldwide (Brockerhoff, Jones, et al., 2006; Fan et al., 2019; Hoch et al., 2020; Nguyen et al., 2024; Rassati et al., 2015; Santoiemma et al., 2024). These trapping protocols allow for both the interception of BWBB introduced via imported commodities (e.g., Rassati et al., 2015; Ruzzier et al., 2023) and the sampling of native species inhabiting the entry points and their surrounding areas (e.g., Mas et al., 2023; Rassati et al., 2018). Such information on native species communities can be crucial, considering that entry sites can also play a key role as sources of potential invaders to other countries connected to those specific sites (Mas et al., 2023).

As with any other surveillance campaign, generic surveillance based on traps can be time‐consuming and labor‐intensive (Epanchin‐Niell, 2017). Several modeling studies have demonstrated that tailoring trapping efforts according to the riskiest entry pathways and/or target taxa phenology can help to increase the cost‐effectiveness of surveillance programs (Epanchin‐Niell et al., 2012, 2014; Yemshanov et al., 2019). However, these theoretical models have not provided practical recommendations for inspectors on how to deploy traps at the points of entry. It is becoming increasingly evident that the composition of the landscape around risk areas should also be considered when planning survey activities (Rassati et al., 2015). Landscape composition is well known to affect insect communities at different spatial scales in both their native and invaded range (Ferrenberg, 2016; Gazzea et al., 2024; Lustig et al., 2017; Rassati, Faccoli, Haack, et al., 2016). For example, the amount of forest cover present in the proximity of a trap (Nunes et al., 2021; Schroeder, 2013) or in the landscape around entry points (Rassati et al., 2015, 2018) was shown to affect the number of native and non‐native BWBB species and individuals collected in traps. Despite these indications, no study has yet investigated whether and how the number and spatial arrangement of the traps used for generic surveillance of BWBB in areas surrounding entry points should be adjusted depending on landscape composition.

**FIGURE 1:**
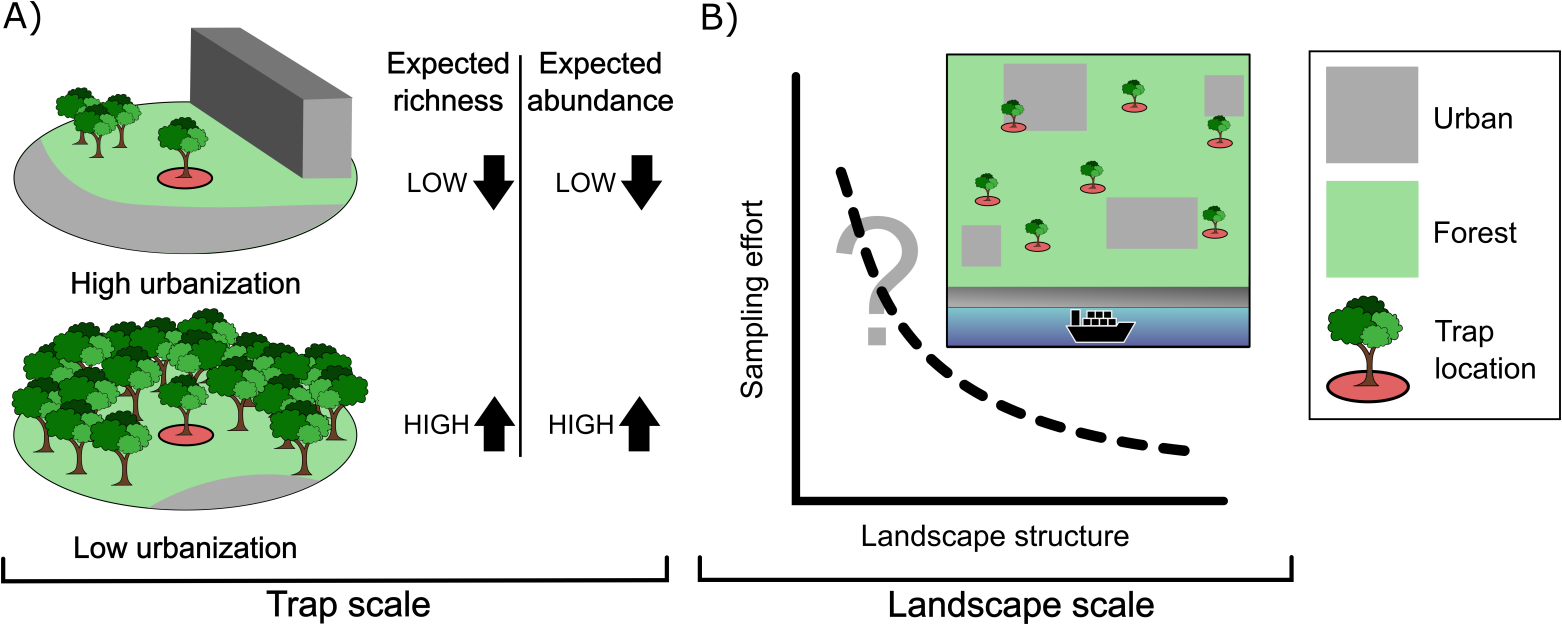
Graphical representation of ecological and practical questions addressed in our study. (A) How the single trap efficacy can be maximized depending on the surroundings at the trap scale; (B) how many traps should be used in a landscape‐wide sampling, depending on the surroundings at the landscape scale. Icons were created by DN using Inkscape software.

Through a trapping experiment carried out in 13 landscapes located around entry points across Europe and North America and selected to span a gradient of landscape composition, this study focused on optimizing trapping protocol efforts for generic surveillance targeting native and non‐native BWBB beetles around high‐risk areas. Among the various taxa of BWBB, we specifically focused on longhorned beetles (Cerambycidae) and bark and ambrosia beetles (Scolytinae) due to methodological constraints related to lure attractiveness and trap type. Specifically, we attempted to address two key questions (Figure 1): (1) how to maximize the single‐trap efficacy depending on the trap surroundings; (2) how many traps should be used in a landscape‐wide sampling for BWBB, depending on the tree cover at the landscape scale. Overall, these results can provide practical information to plant protection agencies on how to optimize trapping protocols for generic surveillance of BWBB around entry points, depending on landscape composition.

## MATERIALS AND METHODS

### Study areas, trap type, and sampling design

**FIGURE 2:**
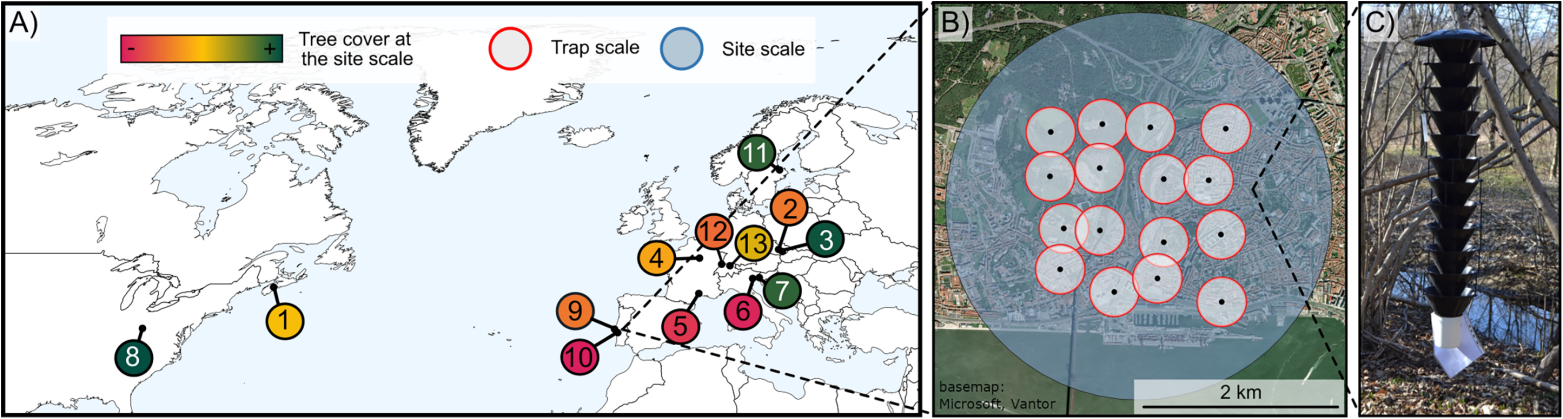
Sampling design of the experiment at multiple scales: (A) locations of the 13 sampled landscapes in Europe and North America, showing the percentage of forest cover at each landscape. The number reported within each colored circle corresponds to the landscape number as in Appendix S1: Table S1. (B) Spatial scales at which the effect of the surrounding habitat was tested: landscape scale (2‐km‐radius buffer from the center of the site) and trap scale (250‐m‐radius buffer around each trap). The background map image is by Esri and used with permission, World Imagery (Source: Esri, DigitalGlobe, GeoEye, i‐cubed, USDA FSA, USGS, AEX, Getmapping, Aerogrid, IGN, IGP, swisstopo, and the GIS User Community). (C) An example of a black understory multi‐funnel trap baited with the multi‐lure blend. Photo credits: Davide Rassati.

The study was conducted at 13 sites across 8 different countries in the temperate zone of Europe (i.e., Czech Republic, France, Italy, Sweden, Switzerland, and Portugal) and North America (i.e., Nova Scotia, Canada, and Ohio, USA) (Figure 2; Appendix S3: Table S1). Selected sites encompassed a gradient of forest cover within a 2‐km buffer, ranging from 10% to 80%. Selected landscapes (i.e., sites) were mostly characterized by mixed forest and urban patches in different proportions. In addition, the sites were all located near high‐risk areas (the distance between the center of sampling area and the high‐risk area was about 1–3 km), such as ports, airports, railway stations, warehouses, and high‐use recreation areas (Appendix S3: Table S1).

At each site, we set up sixteen 12‐unit black funnel traps (Figure 2C) supplied by Synergy Semiochemical Corporation (Delta, BC, Canada). Traps were coated with fluoropolymer suspension to increase trap catches (Allison et al., 2016; Graham & Poland, 2012) and filled with 150–200 mL of either a 50% propylene glycol solution mixed with water, or a pre‐mixed marine/RV antifreeze solution as a preservative and surfactant. More traps were deployed than those previously used in similar surveillance campaigns targeting BWBB (Mas et al., 2023; Rabaglia et al., 2019; Rassati et al., 2015) to test the effect of a sampling effort reduction on the overall sampling efficacy. Traps were set up following a 2 km × 2 km grid (Santoiemma et al., 2024), with one trap in each of the 16 grid cells (0.5 km × 0.5 km each) (Figure 2). In two sites in France, the trap arrangement was based on two transects instead of a square grid. Within the cell, the trap was always set up with the top of the trap about 4–5 m above the ground on lower tree branches, regardless of tree species, to efficiently intercept insects inhabiting the lower forest strata (e.g., Dodds et al., 2010; Flaherty et al., 2019; Ulyshen & Sheehan, 2019). Trees were selected to support the trap weight, positioned as close as possible to the center of each cell, while maintaining a minimum distance of approximately 200 m between traps. Traps were set up in May 2019 and checked and emptied every 2–3 weeks until September of the same year. Preservative liquids were replaced during each trap check.

Traps were baited with a multi‐lure blend, which represents a common practice in generic surveillance protocols (Fan et al., 2019; Roques et al., 2023; Santoiemma et al., 2024, 2025). The multi‐lure blend included cerambycid pheromones, ethanol, and alpha‐pinene and was selected as it was shown to attract a high number of both longhorned beetles and bark and ambrosia beetles (Cavaletto et al., 2020; Roques et al., 2023). An extensive summary of specific chemicals is provided as supplementary (Appendix S1). Trapped BWBB were identified to species level using morphological features and taxonomic keys (list of references for identification in Appendix S2; the complete list of identified species is available as an external dataset in Zenodo: https://doi.org/10.5281/zenodo.17044394, Species_List.xlsx). Then, each species was identified either as native or non‐native to the continent where it was collected. Voucher specimens were deposited in each institution's insect collection. For one site (Sete Pierres Blanches, France), only 15 traps were considered because 1 trap was severely damaged.

### Quantification of the surrounding habitat at the trap scale and landscape composition

At the trap scale, we used a 250 m buffer around each trap (Figure 2). This buffer size was selected based on the results of previous studies testing the attraction range of baited traps towards BWBB (e.g., Dodds & Ross, 2002; Jactel et al., 2019). For each buffer, we computed the tree cover and the angular percentage of the area without artificial barriers such as main roads and buildings. Since we found a high positive correlation between the tree cover and the angular percentage of area without barriers (Pearson's *r* = 0.85), we used a combination of the two variables. We computed an “urbanization” index as the Euclidean distance between the trap variables and a hypothetical “full‐natural” state, meaning 100% tree cover and 100% barrier absence (Appendix S3: Figure S1).

At the landscape scale, we computed the tree cover within a 2 km radius from the center of each of the 13 different sites (Figure 2). Both for the trap scale and for the landscape scale, we used the WorldCover V2 2021 (Zanaga et al., 2022) for tree cover estimation. This dataset is based on Sentinel 2 and Sentinel 1 data and provides global land use at 10‐m resolution. We quantified the tree cover proportion using the landscapemetrics 1.5.5 package (Hesselbarth et al., 2019) in R 4.2.2 (R Core Team, 2024), while barriers were estimated using the Google satellite background image in QGIS 3.34 (QGIS.org, 2024).

### Effect of the surrounding habitat on trap catches at the trap scale

To test the effect of the surrounding habitat at the trap scale on the species richness and abundance of BWBB, we used mixed‐effects models. The species richness and abundance were included in the model as response variables, the urbanization index (standardized values ranging from 0 to 100) as the explanatory variable, and the landscape identity as a random factor. We used Poisson or negative binomial family models to account for non‐normal distributions. The analysis was carried out in R 4.2.2 (R Core Team, 2024), using the packages lme4 1.1‐35.1 (Bates et al., 2015), and lmertest 3.1‐3 (Kuznetsova et al., 2017) for model fitting, dharma 0.4.6 (Harting, 2021) for model diagnostic, and effects 4.2‐2 (Fox & Weisberg, 2019) for model visualization. For this part of the study, we analyzed native and non‐native species separately.

### Effect of landscape composition on turnover and sample coverage

We used beta diversity to test whether the species composition of the BWBB community differed among traps within the same site, and whether the spatial turnover was more or less pronounced depending on the surrounding landscape. For each site, we calculated the average spatial turnover, that is, the replacement component of beta‐diversity (Cardoso et al., 2014; Podani & Schmera, 2011), using binary species × trap matrices. Beta diversity can be decomposed into two components: difference in species richness and spatial turnover. Here, we used spatial turnover, which is twice the number of species in one trap that are replaced by new species in another (Podani & Schmera, 2011). This metric is higher when many species present in one trap are absent from the other, and vice versa, but it does not depend on differences in total species richness between traps. To check whether the traps followed a balanced design depending on the total tree cover at the landscape scale, we compared the tree cover at the trap scale with the overall tree cover at the landscape scale. The average tree cover at the trap scale strongly correlated with the total tree cover at the landscape scale (Pearson's coefficient = 0.81), thus indicating that the traps were deployed in forests and urban areas proportionally across spatial scales. To test the effect of the surrounding landscape on the average species turnover (i.e., replacement component of beta diversity) among trap catches, we used a linear model.

Furthermore, we used incidence data on the presence/absence of each BWBB species across the traps (i.e., sampling units) to estimate the sample completeness (*q* = 0) and sample coverage (*q* = 1) achieved at each landscape based on the number of traps. The sample completeness refers to the proportion of the total site estimated species richness that was observed in the trap samples (observed species/Chao2 species richness estimator). We tested the effect of forest cover on sample completeness at the landscape scale using linear models. The sample coverage represents the probability that a newly recorded incidence, namely a new trap, will only contain species that have already been detected in the current sample (Chao et al., 2014, 2020; Chao & Jost, 2012). The sample coverage was computed for trap number <16 by rarefaction procedure, and for trap number >16 (max = 32) by extrapolation procedure. To test the effect of forest cover on sample coverage at the landscape scale, we used a linear model with the sample coverage at a given trap number (4, 8, 16, and 32) as response variable and the forest cover as explanatory variable. All analyses were done in R 4.2.2 software (R Core Team, 2024) using package bat 2.9 (Cardoso et al., 2015) for beta diversity computation, and inext 3.0 (Hsieh et al., 2016) and inext.4.steps 1.0 (Chao et al., 2020) for estimating the sample completeness/coverage. For these analyses, we used the entire community of BWBB without distinguishing between native and non‐native species.

### Effect of sampling effort reduction

To investigate the effect of sampling effort reduction (i.e., number of traps) on trap catches, we simulated multiple scenarios by gradually reducing the number of traps at each landscape. These scenarios included random removal and sequential removal according to the urbanization index (i.e., ascending and descending order). For random removal, we performed 100 randomizations. At each step, corresponding to the sequential removal of one trap, we calculated the percentage of species lost in the subsamples (i.e., the number of species excluded from the total pool due to trap removal). We used the percentage to remove the effect of different species richness among the landscapes. We then compared the percentage of species loss when sampling effort was reduced by 50% (i.e., 8 traps per landscape) across different methods. Because many trapping programs for surveillance of non‐native BWBB deploy fewer than eight traps per landscape (e.g., three in Rabaglia et al., 2019; six in Thurston et al., 2022), we also computed the percentage of species loss after reducing sampling effort by 75% (4 traps per landscape). Comparisons were done in R 4.2.2 software (R Core Team, 2024) using emmeans 1.8 (Lenth et al., 2020). For this analysis, we used the entire community of BWBB without distinguishing between native and non‐native species.

Finally, we used a linear model to test the effect of the surroundings at the landscape scale (i.e., tree cover) on the percentage of species loss when reducing the traps from 16 to 8 and to 4, respectively, under a random removal scenario. The analysis was carried out in R 4.2.2 software (R Core Team, 2024) using dharma 0.4.6 (Harting, 2021) for model diagnostics and effects 4.2‐2 (Fox & Weisberg, 2019) for model visualization. For this analysis, we used the entire community of BWBB without distinguishing between native and non‐native species. R scripts and datasets are available as an external dataset in Zenodo: https://doi.org/10.5281/zenodo.17044394.

## RESULTS

### General results

Overall, we collected 15,072 longhorned beetles (167 species), 8416 bark beetles (105 species), and 38,267 ambrosia beetles (27 species). In Europe, the most abundant native species for each taxon were the ambrosia beetle *Xyleborinus saxesenii* (28,332 individuals), the bark beetle *Orthotomicus erosus* (3816 individuals), and the longhorned beetle *Phymatodes testaceus* (3511 individuals). In North America, the most abundant native species were the ambrosia beetle *Anisandrus sayi* (989 individuals), the longhorned beetle *Phymatodes amoenus* (732 individuals), and the bark beetle *Polygraphus rufipennis* (474 individuals). Despite most of the collected species being native (93%, 287 species), 21 species were non‐native. In Europe, we collected four non‐native longhorned beetles, that is, *Neoclytus acuminatus*, *Phoracantha recurva*, *Xylotrechus chinensis*, and *Xylotrechus stebbingi*, two non‐native bark beetles, that is, *Cyrtogenius luteus* and *Hypothenemus eruditus*, and five non‐native ambrosia beetles, that is, *Amasa parviseta*, *Ambrosiophilus atratus*, *Gnathotrichus materiarius*, *Xylosandrus crassiusculus*, and *Xylosandrus germanus*. In North America, we collected one non‐native longhorned beetle, that is, *P. testaceus*, two non‐native bark beetles, that is, *Crypturgus pusillus* and *Hylastes opacus*, and nine non‐native ambrosia beetles, that is, *A. atratus*, *Anisandrus maiche*, *Cnestus mutilatus*, *Dryoxylon onoharaense*, *Euwallacea validus*, *Xyleborinus attenuatus*, *X. saxesenii*, *X. crassiusculus*, and *X. germanus*.

### Effect of the surrounding habitat at trap scale

**FIGURE 3:**
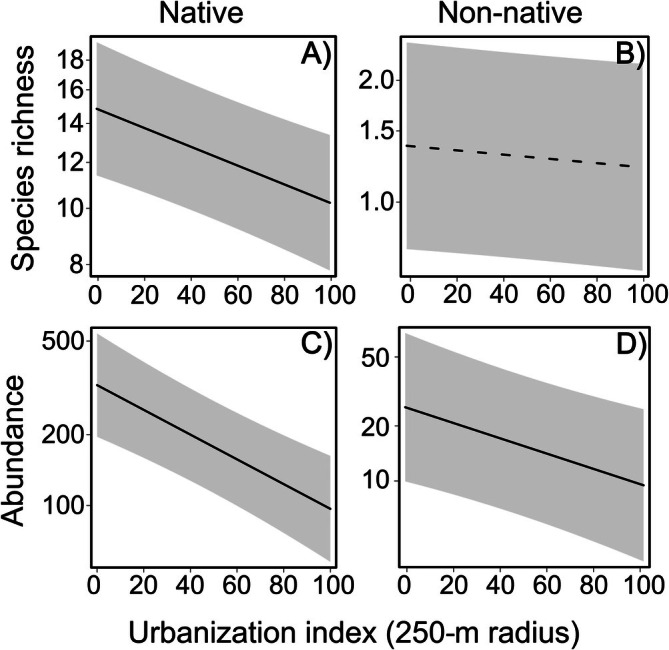
Effect of urbanization index at the trap scale (250‐m‐radius buffer) on species richness and abundance of native (A and C, respectively) and non‐native species (B and D, respectively). Solid lines and dashed lines indicate significant and non‐significant trends, respectively. Gray areas indicate CIs (95%) for regressions. Urbanization index is defined as the Euclidean distance of tree cover and presence of barriers within a 250‐m buffer from the natural condition (full tree cover and absence of barriers). Urbanization index increases along urbanization gradient, capturing both a decrease in resources and an increase in structural impediments to beetle movements through the landscape.

Both species richness and abundance of native species were significantly affected by the urbanization index computed at the 250‐m buffer around the traps (species richness: generalized mixed‐effects model, χ^2^ = 26.51, *p* < 0.0001; abundance: negative binomial mixed‐effects model, χ^2^ = 32.54, *p* < 0.0001). Both values increased with decreasing value of the urbanization index (Figure 3A,C). For non‐native species, we found a similar effect of the urbanization index on abundance (negative binomial mixed‐effects model, χ^2^ = 7.91, *p* = 0.005) but not on species richness (generalized mixed‐effects model, χ^2^ = 2.19, *p* = 0.139) (Figure 3B,D).

### Effect of landscape composition on turnover and sample coverage

**FIGURE 4:**
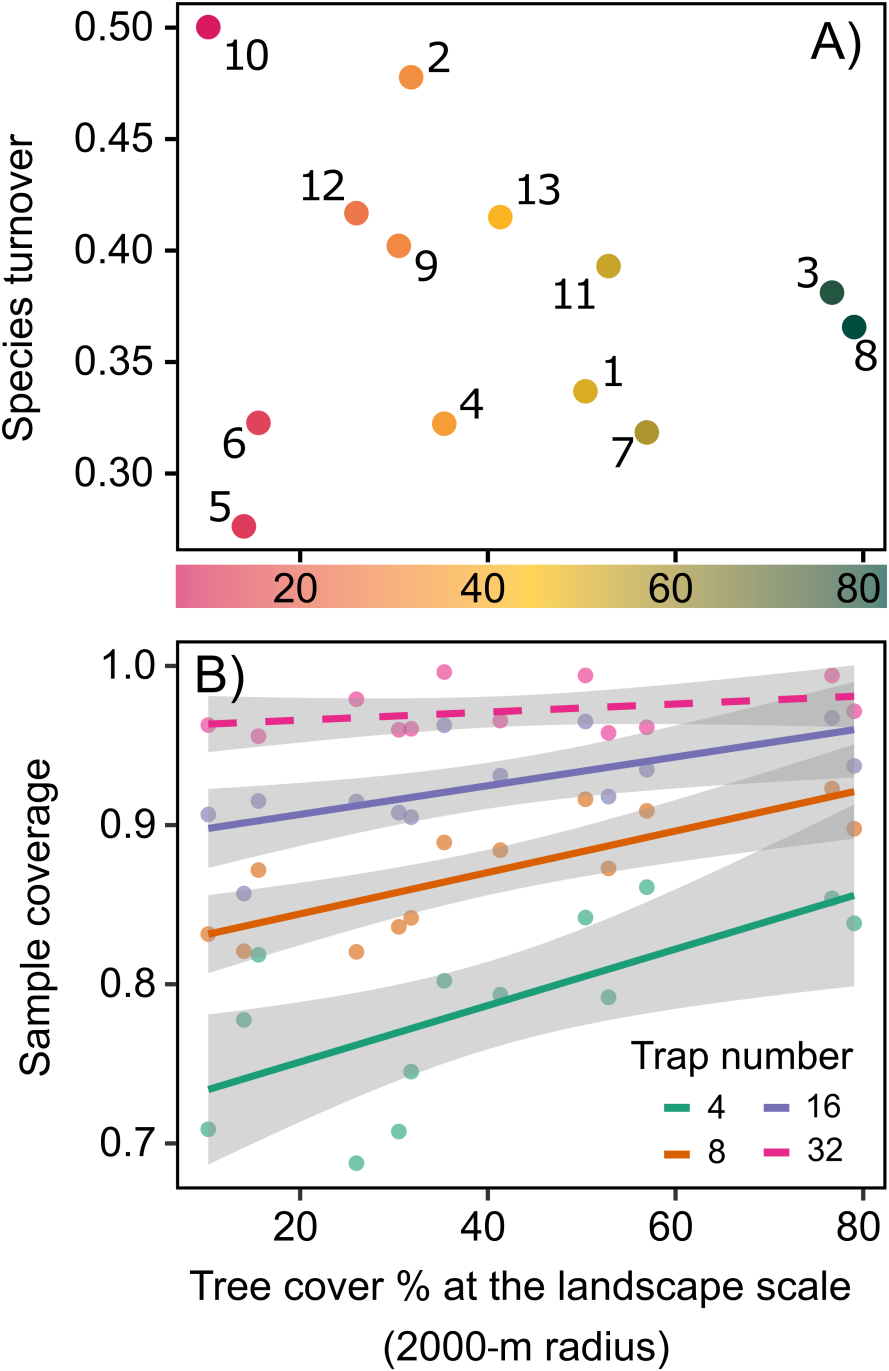
Effects of tree cover at the landscape scale on species turnover and sample coverage. (A) Species turnover among trap catches averaged at the landscape scale for each of the 13 entry sites where traps were set up. The number reported near each colored circle corresponds to the site IDs as in Appendix S1: Table S1 and Figure 1. The color of the circle represents the tree cover calculated at the landscape scale. (B) Effect of tree cover at the landscape scale on sample coverage under different numbers of traps (4, 8, 16, 32). Solid lines indicate significant regressions, dashed lines indicate non‐significant regressions. Gray areas indicate CIs (95%) for regressions.

Overall, the average species turnover among the traps within the same landscape was 0.38 (min: 0.28; max: 0.50) (Figure 4A). This value was not affected by the tree cover at the landscape scale (Linear regression, *F*_1,11_ = 0.25, *p* = 0.634). For each landscape, we measured the observed species number, estimated richness, sample completeness, and sample coverage (Appendix S3: Table S2). We found that the average sample completeness (proportion of estimated total species richness observed in traps) was 71% (min: 34%; max: 92%), but the sample completeness was not affected by tree cover at the landscape scale (Linear regression, *F* = 1.55, *p* = 0.239). The average sample coverage was 0.93 (min: 0.86; max: 0.97). This value was affected by the tree cover depending on the number of traps (Linear model, for 4 traps: *F*_1,11_ = 9.24, *R* ^2^ = 0.41, *p* = 0.011; for 8 traps: *F*_1,11_ = 18.44, *R* ^2^ = 0.59, *p* = 0.001; for 16 traps: *F*_1,11_ = 8.65, *R* ^2^ = 0.40, *p* = 0.013; for 32 traps (extrapolated): *F*_1,10_ = 1.50, *R* ^2^ = 0.44, *p* = 0.248, Figure 4B). We did not find a significant effect of species richness on sample coverage; thus, our data were consistent across landscapes.

### Effect of sampling effort reduction

**FIGURE 5:**
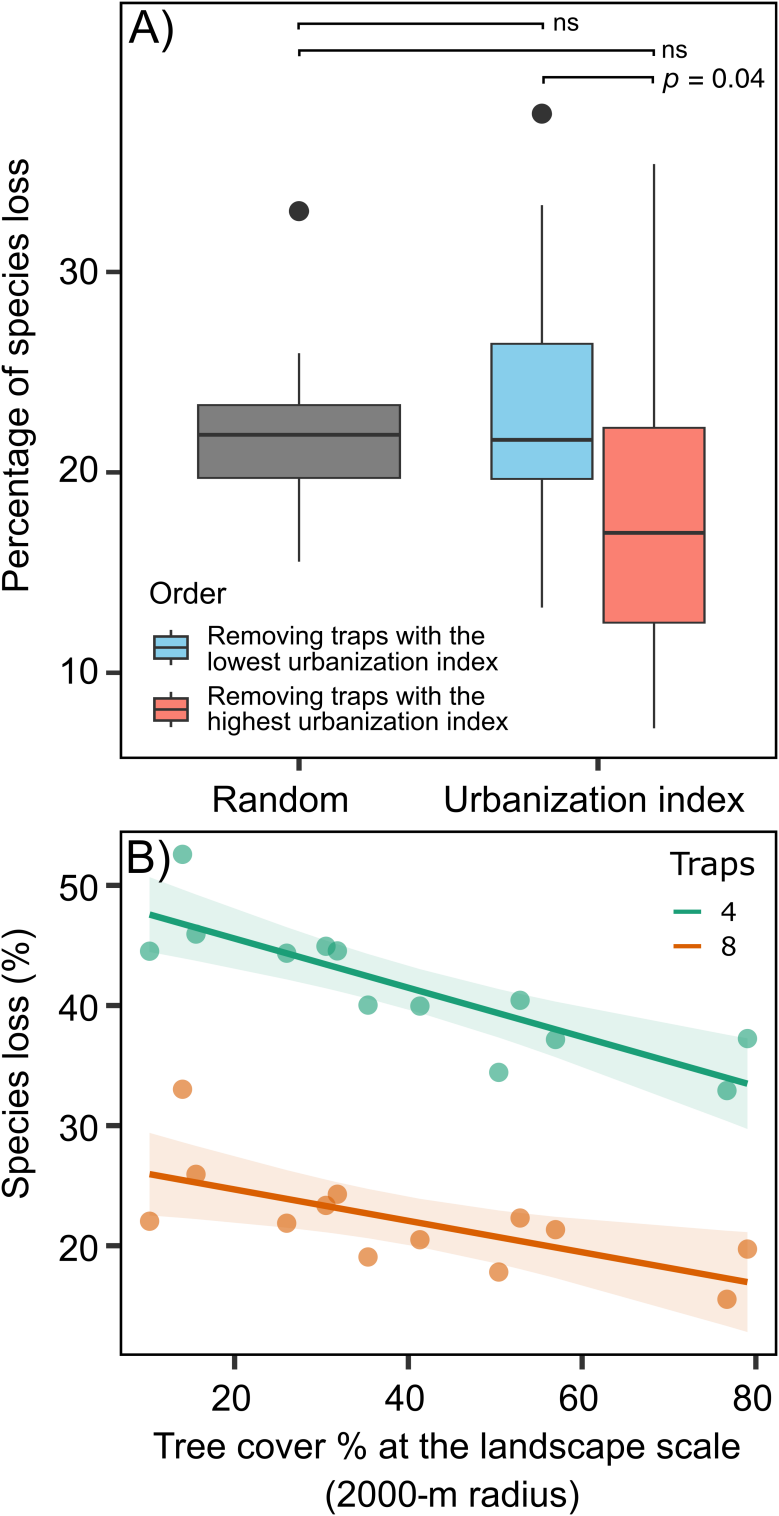
Percentage of species loss (i.e., percentage of species that are lost when traps are removed) was simulated for 8 and 4 traps (reducing factors 2 and 4, respectively) under different scenarios: Random trap removal and ordered trap removal depending on the urbanization index. (A) Boxplots with 8 traps comparing random trap removal (i.e., gray boxplot) and ascending order of urbanization (i.e., blue boxplot), and descending order of urbanization (i.e., red boxplot). In the ascending order, traps with a lower urbanization index were removed first, while in the descending order, traps with a higher urbanization index were removed first. *p* values of pairwise comparisons are reported on top (“ns” for non‐significant differences). (B) Effect of tree cover at the site scale on the proportion of species loss when reducing the sampling effort from 16 to 8 traps (orange line) and from 16 to 4 traps (green line). Gray areas indicate CIs (95%) for regressions.

The average species loss with halved sampling effort (i.e., from 16 to 8 traps) was 22.5% for random removal, 23.6% for ascending urbanization index (traps with higher urbanization index were removed first), and 17.7% for descending urbanization index (traps with lower urbanization index were removed first) (Figure 5A; Appendix S3: Figures S3 and S4). Under these different scenarios, we found significant differences in the percentage of species loss using different methods (ANOVA, χ^2^ = 7.08, *p* = 0.029). Although the average species loss between random removal and gradient‐driven removal in any order was not significantly different (contrasts: random vs. ascending urbanization index, *p* = 0.772; random vs. descending urbanization index, *p* = 0.166), we found that fewer species were lost when traps were removed from the higher urbanization index to the lower, compared to the inverse order (estimated marginal means contrast: ascending urbanization index vs. descending urbanization index, *p* = 0.043). Although we did not find differences among different scenarios with 4 traps (75% reduced sampling effort), the mean percentage in species loss was high: 41.5% for the random scenario, 43.7% when removing traps with a lower urbanization index first, and 40.7% when removing traps with a higher urbanization index first.

Tree cover at the landscape scale (i.e., 2‐km‐radius buffer) significantly affected the percentage of species loss with random removal when sampling effort is reduced both from 16 to 8 traps (Linear regression, *F*_1,11_ = 9.46, *R* ^2^ = 0.41, *p* = 0.011), and from 16 to 4 traps (Linear regression, *F*_1,11_ = 28.26, *R* ^2^ = 0.69, *p* < 0.001). In particular, when reducing the number of traps to 8 and 4, the percentage of species loss decreased with increasing tree cover at the landscape scale (Figure 5B), that is, urban‐dominated landscapes were more sensitive than forest‐dominated landscapes to sampling effort reduction.

## DISCUSSION

Semiochemical‐baited traps are a key component of generic post‐border surveillance aimed at detecting both native and non‐native species in areas surrounding entry sites (Dodds et al., 2024; Mas et al., 2023). Survey programs are often constrained by budget, which limits the capacity of plant protection agencies to intensively monitor high‐risk areas and their surroundings. Although previous studies investigated potential approaches to optimize sampling efforts among surveillance locations (Blackburn et al., 2017; Koch et al., 2011; Yemshanov et al., 2010a, 2010b, 2019), there has been no investigation of whether and/or how the number and position of the traps used for generic surveillance of BWBB in areas surrounding entry points should be adjusted depending on landscape composition. Through this multi‐country trapping study carried out in Europe and North America, we showed that integrating landscape ecology in plans for generic BWBB surveillance in areas surrounding entry points can help optimize trap location across the landscape. This approach can maximize the number of BWBB species that can be potentially collected in traps, aiding surveillance at both national and international scales.

We found that trap catches depend on trap surroundings. In particular, the abundance of both native and non‐native species and the richness of native species increased as the urbanization index decreased. We did not find a significant effect on species richness of non‐native species, likely because very low numbers of non‐native species were collected during the study. The effect of the surrounding habitat might be due to two non‐mutually exclusive mechanisms related to the features that we considered: the amount of forest cover and the amount of barriers to insect dispersal present in the trap surroundings. Because these two variables are usually strongly correlated, these processes could not be tested independently. Regarding the effect of tree cover, BWBB can be negatively affected by the decrease in resource availability (i.e., habitat loss) (Baur et al., 2020; Li et al., 2020; Park & Reid, 2007; Saint‐Germain & Drapeau, 2011; Vitali et al., 2023). Rassati et al. (2018) found that both species richness and abundance of BWBB increased with increasing tree cover in heterogeneous landscapes. Regarding the effect of barriers, insect dispersal across the landscape can be limited by the presence of roads and buildings in anthropized areas (Andersson et al., 2017), or even by patches of non‐host tree species (Jones et al., 2019; Nunes et al., 2021). Consequently, traps surrounded by many anthropogenic barriers are more likely to be approached by fewer species and individuals than traps surrounded by highly connected forest habitat patches.

Considering the whole landscape, our sampling protocol (16 traps in a 2 × 2 km grid) showed an average sample completeness of 72%, meaning that almost 30% of total species richness was not detected. Although the species richness estimator is extremely sensitive to very rare species, one site showed a sample completeness lower than 50%, suggesting that under some conditions (high urbanization), even 16 traps did not catch a satisfactory number of species. We also found some turnover among the BWBB species collected in traps in all landscapes, even though we did not find any significant effect of tree cover. This pattern is very common when comparing catches obtained with traps spread across a landscape because the collected species and the related number of individuals can be affected by many variables or stochasticity (Dodds et al., 2024; Sweeney et al., 2020). For example, the species identity and the health status of the trees nearby can be particularly relevant for traps set up in heterogeneous landscapes (Gatti et al., 2018; Marchioro et al., 2022; Meng et al., 2013; Rassati, Faccoli, Battisti, & Marini, 2016; Ulyshen & Hanula, 2007). Similarly, the amount of sunlight reaching the trap might affect the release rate of semiochemicals from lures and consequently increase or decrease trap catches (Nielsen et al., 2019).

Analysis of sample coverage generally confirmed the same conclusion but also showed that using a higher number of traps becomes particularly important in urban‐dominated landscapes. Urban areas may offer a higher diversity of potential tree hosts than natural areas and represent the most common sites where non‐native BWBB species are first detected (Augustinus et al., 2025; Branco et al., 2019). The higher resource variability in urban areas can increase the spatial variability of BWBB communities, thus calling for a higher sampling effort in urban‐dominated landscapes. This criterion was confirmed when simulating a reduction in the number of traps used in the different landscapes. A 50% reduction (i.e., from 16 to 8 traps) caused the loss of an increasing percentage of species with decreasing tree cover at the landscape scale, indicating that entry points in urban landscapes would require allocating more traps. Overall, our simulation showed that reducing the number of traps by 50% led to an approximately 20% decrease in collected BWBB species. Previous studies on Scolytinae in tree plantations showed ~10% species loss with halved sampling effort (Haack et al., 2025). Although landscape composition affects sampling effort at a larger scale, the surrounding habitat at the trap scale also influences catches. Despite a weak effect, the contrasting trap removal scenarios revealed a higher contribution for those traps showing a lower urbanization index, similarly to the effect on trap catches at the trap scale. These findings suggest that prioritizing trap locations characterized by high tree cover and few anthropogenic barriers at the trap scale (i.e., low urbanization index) would help to maximize the number of collected species, given a limited availability of traps.

One limitation of our study is that we could draw separate conclusions for native and non‐native species only when evaluating how the surroundings at the trap scale influenced trap catch. Future research should aim to explore other potential differences between native and non‐native species, as this could suggest that different strategies are needed for each. Additionally, our findings are only valid for those BWBB attracted by the trapping method we used. Future studies should further investigate how the landscape impacts landscape‐wide sampling of various BWBB taxa. To do this, different trap colors (Besana et al., 2025; Cavaletto et al., 2021; Santoiemma et al., 2025) or more complex attractive blends could be tested. Similarly, traps should be placed at multiple heights to sample beetle communities inhabiting different forest layers (Dodds et al., 2024).

### Implications for management

Generic surveillance based on baited traps is becoming increasingly important for BWBB because it provides opportunities to simultaneously monitor non‐native species entering the country and native species that can potentially become invaders in other countries. Here, we showed that tailoring the number of traps and their location based on the surrounding habitat may help to optimize resources and overall efforts. This study provides helpful criteria when planning generic surveillance around risk areas located in heterogeneous landscapes. First, plant protection agencies should allocate a higher number of traps in entry points surrounded by urban‐dominated landscapes, while a lower sampling effort may be required in forest‐dominated landscapes. Based on our results and considering a 4 km^2^ surveyed area, we suggest using at least 8 traps in forest‐dominated landscapes (80% tree cover) and increasing this number to 16 in high urbanized landscapes (<10% tree cover) to ensure at least 80% of sample coverage. In case of budget constraints that limit the number of traps, the best locations to set up the traps should be based on their surroundings (trap scale features). In particular, to maximize the overall trap efficacy, the single traps should be preferentially set up near available forest patches or urban parks, while locations with high presence of roads and buildings in the surroundings should be avoided. However, we stressed that using fewer than 8 traps does not allow a good representation of BWBB diversity occurring at the landscape scale. Since sample coverage did not vary with species richness, these findings could be extended to temperate Europe and North America. However, several challenges remain in the routine use of these generic surveillance programs by phytosanitary services: for example, the need for faster and more accurate specimen identification (e.g., AI‐based automated recognition) and digital innovations (e.g., automated traps) to decrease the amount of time spent manually checking traps.

## AUTHOR CONTRIBUTIONS

**Davide Nardi:** Conceptualization; data curation; formal analysis; visualization; writing—original draft; writing—review and editing. **Davide Rassati:** Conceptualization; data curation; project administration; validation; visualization; writing—original draft; writing—review and editing. **Andrea Battisti:** Funding acquisition, writing—review and editing. **Manuela Branco:** Funding acquisition, writing—review and editing. **Claudine Courtin:** Lure preparation; methodology. **Massimo Faccoli:** Beetle identification, writing—review and editing. **Nina Feddern:** Investigation. **Joseph A. Francese:** Funding acquisition; investigation; writing—review and editing. **Emily Franzen:** Beetle identification, investigation. **André Garcia**: Writing—review and editing, investigation. **Filippo Giannone:** Beetle identification. **Martin M. Gossner:** Funding acquisition, investigation. **Mats Jonsell:** Beetle identification, investigation, writing—review and editing. **Chantelle Kostanowicz:** Beetle identification, investigation. **Matteo Marchioro:** Investigation, writing—review and editing. **Petr Martinek:** Beetle identification, investigation. **Ann M. Ray:** Funding acquisition, investigation, writing—review and editing. **Alain Roques:** Investigation, beetle identification, funding acquisition. **Jon Sweeney:** Funding acquisition, investigation, writing—review and editing. **Kate Van Rooyen:** Beetle identification, investigation. **Vincent Webster:** Beetle identification, investigation. **Lorenzo Marini:** Conceptualization, formal analysis, writing—original draft, writing—review and editing. All authors approved the text.

## FUNDING INFORMATION

This work was supported by the European Union's Horizon 2020 Research and Innovation Program under grant agreement 771271 “HOMED – Management of Emerging Forest Pests and Diseases.” D. Nardi was partially supported by the European Union's Horizon Europe Research and Innovation Programme under grant agreement 101134200 “Forest surveillance with artificial intelligence and digital technologies – FORSAID.” Manuela Branco and André Garcia were also supported by FCT – Fundação para a Ciência e a Tecnologia, I.P., Project UID/00239: Centro de Estudos Florestais. J Sweeney was supported by the United States Department of Agriculture Animal and Plant Health Inspection Service Cooperative Agreement AP18PPQS&T00C164 (PPA 7721 Project – FY18 3.0185.01) and Natural Resources Canada, Canadian Forest Service A‐base Forest Pest Risk Management Program. Ann M. Ray and Emily Franzen were supported by cooperative agreements between the USDA APHIS PPQ S&T and Xavier University: AP18PPQS&T00C169 and AP19PPQS&T00C082.

## CONFLICT OF INTEREST STATEMENT

The authors declare no conflicts of interest.

## Supporting information


Appendix S1.

Appendix S2.

Appendix S3.

## Data Availability

Data and code (Nardi, 2026) are available in Zenodo at https://doi.org/10.5281/zenodo.17044394.
