# Supplementary material for: Integrating landscape ecology into generic surveillance plans for bark‐ and wood‐boring beetles
Source: Ecol Appl. 2026 Mar 9;36(2):e70194. doi: 10.1002/eap.70194 (PMC12968951; doi:10.1002/eap.70194)
Supplement: Supplementary file 1 — Appendix S1. [file EAP-36-e70194-s002.pdf]

SUPPORTING INFORMATION

Integrating landscape ecology into generic surveillance plans for bark- and wood-boring beetles

Davide Nardi, Davide Rassati, Andrea Battisti, Manuela Branco, Claudine Courtin, Massimo Faccoli, Nina Feddern, Joseph A. Francese, Emily Franzen, André Garcia, Filippo Giannone, Martin M. Gossner, Mats Jonsell, Chantelle Kostanowicz, Matteo Marchioro, Petr Martinek, Ann M. Ray, Alain Roques, Jon Sweeney, Kate Van Rooyen, Vincent Webster, Lorenzo Marini

APPENDIX S1: Multi-lure blends – Materials and Methods

Attractive lures used to bait the traps

For the multi-lure blends for black traps in our experiment, we followed the methods described in Santoiemma et al. (2024). Briefly, we used a blend of eight longhorn beetle pheromones (racemic fuscumol (volume amount: 50 mg), racemic fuscumol acetate (50 mg), geranyl acetone (25 mg), racemic 3-hydroxyhexan-2-one (50 mg), prionic acid (1 mg), 2-methylbutan-1-ol (50 mg), *anti*-2,3-hexanediol (50 mg), and monochamol (50 mg)), adding also UHR (ultra-high release rate) ethanol and alpha-pinene lures. Longhorn beetle pheromones are attractive for a wide range of subfamilies (Fan et al., 2019; Roques et al., 2023; Santoiemma et al., 2025). The host volatile lures (i.e., ethanol and alpha-pinene) were added to the pheromone blend because they increase the attractiveness of the trap to many species of longhorn beetles (Miller et al., 2015; Collignon et al., 2016) and bark and ambrosia beetles (Miller & Rabaglia, 2009; Marchioro et al., 2020). All compounds were dissolved in isopropanol as a carrier to a total volume of 1 ml per lure. Release rates are specified in Fan et al. (2019). One-milliliter aliquots of the pheromone blend were stored at 4 °C until usage. At the beginning of the experiment and during each trap check, the 1 ml aliquots were poured into a clear polyethylene sachet containing a cotton cylinder, which was hung on the trap using a string or plastic cable tie. All pheromones were purchased from ChemTica Internacional (Santo Domingo, Costa Rica) except prionic acid (Alpha Scents Inc., West Linn, Oregon, USA); the ethanol and alpha-pinene lures were provided by Econex (Spain).

References

Collignon, R. M., Swift, I. P., Zou, Y., McElfresh, J. S., Hanks, L. M., & Millar, J. G. (2016). The influence of host plant volatiles on the attraction of longhorn beetles to pheromones. *Journal of Chemical Ecology*, 42, 215-229. https://doi.org/10.1007/s10886-016-0679-x

Fan, J. T., Denux, O., Courtin, C., Bernard, A., Javal, M., Millar, J. G., Hanks, L. M., & Roques, A. (2019). Multi-component blends for trapping native and exotic longhorn beetles at potential points-of-entry and in forests. *Journal of Pest Science*, 92, 281-297. https://doi.org/10.1007/s10340-018-0997-6

Marchioro, M., Rassati, D., Faccoli, M., Van Rooyen, K., Kostanowicz, C., Webster, V., Mayo, P., & Sweeney, J. (2020). Maximizing bark and ambrosia beetle (Coleoptera: Curculionidae) catches in trapping surveys for longhorn and jewel beetles. *Journal of Economic Entomology*, 113, 2745-2757. https://doi.org/10.1093/jee/toaa181

Miller, D. R., Crowe, C. M., Dodds, K. J., Galligan, L. D., De Groot, P., Hoebeke, E. R., Mayfield III, A. E., Poland, T. M., Raffa, K. F., & Sweeney, J. D. (2015). Ipsenol, ipsdienol, ethanol, and α-pinene: trap lure blend for Cerambycidae and Buprestidae (Coleoptera) in pine forests of eastern North America. *Journal of Economic Entomology*, 108, 1837-1851. https://doi.org/10.1093/jee/tov126

Miller, D. R., & Rabaglia, R. J. (2009). Ethanol and (−)-α-pinene: Attractant kairomones for bark and ambrosia beetles in the southeastern US. *Journal of Chemical Ecology*, 35, 435-448. https://doi.org/10.1007/s10886-009-9613-9

Roques, A., Ren, L., Rassati, D., Shi, J., Akulov, E., Audsley, N., Auger-Rozenberg, M.-A., Avtzis, D., Battisti, A., Bellanger, R., Bernard, A., Bernadinelli, I., Branco, M., Cavaletto, G., Cocquempot, C., Contarini, M., Courtial, B., Courtin, C., Denux, O., Dvořák, M., et al. (2023). Worldwide tests of generic attractants, a promising tool for early detection of non-native cerambycid species. *NeoBiota,* 84, 169-209. https://doi.org/10.3897/neobiota.84.91096

Santoiemma, G., Battisti, A., Courtin, C., Curletti, G., Faccoli, M., Feddern, N., Francese, J. A., Franzen, E. K. L., Giannone, F., Gossner, M. M., Kostanowicz, C., Marchioro, M., Nardi, D., Ray, A. M., Roques, A., Sweeney, J., Rooyen, K. V., Webster, V., & Rassati, D. (2024). Testing a trapping protocol for generic surveillance of wood-boring beetles in heterogeneous landscapes. *NeoBiota,* 95, 77–95. https://doi.org/10.3897/neobiota.95.129483

Santoiemma, G., Sweeney, J., Booth, E. G., Cavaletto, G., Curletti, G., Devine, S. M., Francese, J. A., Franzen, E. K. L., Giannone, F., Giasson, M., Gutowski, J. M., Hughes, C., Kimoto, T., Kostanowicz, C., Mokrzycki, T., Plewa, R., Ray, A. M., Qingfan, M., Williams, D., Yan, L., & Rassati, D. (2025). Efficacy of unbaited and baited green multi-funnel traps for detection of *Agrilus* species and other wood-boring beetle taxa. *Journal of Pest Science* https://doi.org/10.1007/s10340-024-01865-z
