# Supplementary material for: Integrating landscape ecology into generic surveillance plans for bark‐ and wood‐boring beetles
Source: Ecol Appl. 2026 Mar 9;36(2):e70194. doi: 10.1002/eap.70194 (PMC12968951; doi:10.1002/eap.70194)
Supplement: Supplementary file 2 — Appendix S2. [file EAP-36-e70194-s001.pdf]

SUPPORTING INFORMATION

Integrating landscape ecology into generic surveillance plans for bark- and wood-boring beetles

Davide Nardi, Davide Rassati, Andrea Battisti, Manuela Branco, Claudine Courtin, Massimo Faccoli, Nina Feddern, Joseph A. Francese, Emily Franzen, André Garcia, Filippo Giannone, Martin M. Gossner, Mats Jonsell, Chantelle Kostanowicz, Matteo Marchioro, Petr Martinek, Ann M. Ray, Alain Roques, Jon Sweeney, Kate Van Rooyen, Vincent Webster, Lorenzo Marini

APPENDIX S2: Taxonomical reference for identified specimens

Specimens have been identified using Ehnström and Holmer (2007) and Bense (1995) for European longhorn beetles; Balachowsky (1949), Grune (1979), and Pfeffer (1995) for European bark and ambrosia beetles; Yanega (1996), Lingaferter (2008), and Bousquet et al. (2017) for North American longhorn beetles; Bright et al. (1976) and Rabaglia et al. (2009) for North American bark and ambrosia beetles. The complete list of species can be found in the external repository (https://doi.org/10.5281/zenodo.17044394).

References

Balachowsky, A., 1949. *Coléoptères Scolytides*. Faune de France, 50. Paris, Lechevalier.

Bense, U., 1995. *Longhorn beetles: illustrated key to the Cerambycidae and Vesperidae of Europe*, Margraf, 512 pp.

Bousquet, Y., Laplante, S., Hammond, H.E.J., Langor, D.W., 2017. *Cerambycidae (Coleoptera) of Canada and Alaska: Identification guide with nomenclatural, taxonomic, distributional, host-plant, and ecological data*. Prague, Nakladatelství Jan Farkač, 300 pp.

Bright, D.E., 1976 *The bark beetles of Canada and Alaska: Coleoptera, Scolytidae*. In The Insects and Arachnids of Canada, Part 2; Canadian Government Publishing: Ottawa, ON, Canada, 1241 pp.

Ehnström, B. & Holmer, M., 2007. *Skalbaggar: Långhorningar. Coleoptera: Cerambycidae*. Nationalnyckeln till Sveriges Flora och Fauna, ArtDatabanken, SLU, Uppsala.

Grüne, S., 1979. *Brief illustrated key to European bark beetles*. Hannover, Germany, M. and H. Schaper.

Lingaferter, S.W., 2008, *Illustrated Key to the Longhorned Woodboring Beetles of the Eastern United States*, Coleopterists Society, North Potomac.

Pfeffer, A., 1995. *Zentral- und westpaläarktische Borken- und Kernkäfer* (Coleoptera: Scolytidae, Platypodidae). Basel, Switzerland, Pro Entomologia, Naturhistorisches Museum.

Rabaglia, R., Dole, S., Cognato, A., 2009. *Review of American Xyleborina (Coleoptera: Curculionidae: Scolytinae) Occurring North of Mexico, with an Illustrated Key*. Annals of the Entomological Society of America. 99. 1034-1056.

Yanega, D., 1996. *Field guide to northeastern longhorned beetles (Coleoptera: Cerambycidae)*. Illinois Natural History Survey Manual 6, 174 pp.
