# Supplementary material for: Integrating landscape ecology into generic surveillance plans for bark‐ and wood‐boring beetles
Source: Ecol Appl. 2026 Mar 9;36(2):e70194. doi: 10.1002/eap.70194 (PMC12968951; doi:10.1002/eap.70194)

SUPPORTING INFORMATION

Integrating landscape ecology into generic surveillance plans for bark- and wood-boring beetles

Davide Nardi, Davide Rassati, Andrea Battisti, Manuela Branco, Claudine Courtin, Massimo Faccoli, Nina Feddern, Joseph A. Francese, Emily Franzen, André Garcia, Filippo Giannone, Martin M. Gossner, Mats Jonsell, Chantelle Kostanowicz, Matteo Marchioro, Petr Martinek, Ann M. Ray, Alain Roques, Jon Sweeney, Kate Van Rooyen, Vincent Webster, Lorenzo Marini

APPENDIX S3: Supplementary Tables and Figures

Table S1. Indicative coordinates of the sites where traps were set up for each country involved in the study. The type of selected high-risk areas in the proximity of which traps were set up is also reported. Sixteen black-understory multi-funnel traps were employed at each site.

Country	Landscape ID	Site name	High-risk area	Latitude (WGS84; decimal degrees)	Longitude (WGS84; decimal degrees)
Czechia	2	Brno (city)	Railway station	49.22	16.61
	3	Brno (hill)	Railway station	49.23	16.68
France	4	Le Sausset	Airport	48.58	02.30
	5	Les Pierres Blanches	Seaport	43.24	03.40
Italy	6	Mestre	Seaport	45.29	12.16
	7	Monfalcone	Seaport	45.48	13.33
Portugal	9	Lisbon	Seaport	38.71	- 9.17
	10	Setubal	Seaport	38.51	- 8.85
Sweden	11	Hargshamn	Seaport	60.15	18.48
Switzerland	12	Basel	River port	47.34	07.36
	13	Zurich	Airport	47.27	08.35
Canada	1	Halifax (Nova Scotia)	Warehouses where containers from the nearby port are unloaded	44.43	- 63.35
USA	8	Cincinnati (Ohio)	High-use recreation area near a campground	39.02	- 84.07

Table S2: For each landscape, we summarized the observed species richness, the Shannon index, the sample completeness (observed species / Chao2 species richness estimator), and the sample coverage (probability that a new incidence data would contain only species previously found). Sample completeness and sample coverage are reported along with 95% confidence intervals based on a bootstrap procedure (n = 100).

Site name /landscape	Trap numbers	Observed richness	Incidence-based Shannon index	Sample completeness	s.e.	Sample coverage	s.e.
Basel	16	48	32.6	0.815	0.086	0.915	0.018
Cincinnati (Ohio)	16	61	40.3	0.702	0.095	0.937	0.011
Halifax (Nova Scotia)	16	73	52.1	0.896	0.06	0.965	0.007
Hargshamn	16	64	42.1	0.64	0.109	0.918	0.013
Le Sausset	16	32	21	0.918	0.11	0.963	0.015
Lisbon	16	28	19.7	0.705	0.146	0.908	0.025
Brno (city)	16	66	43.3	0.696	0.096	0.905	0.015
Brno (hill)	16	83	57.6	0.891	0.068	0.967	0.007
Mestre	16	37	21.7	0.607	0.144	0.915	0.018
Monfalcone	16	53	34.7	0.586	0.153	0.935	0.013
Les Pierres Blanches	15	36	19.7	0.344	0.15	0.854	0.024
Setubal	16	25	17.6	0.725	0.136	0.907	0.028
Zurich	16	62	41.1	0.677	0.106	0.931	0.014

Figure S1. Graphical representation of the urbanization index that we used to test the effect of the surroundings at the trap scale. The urbanization index is defined as the Euclidean distance from the maximum naturality condition (N), which is defined as 100% forest cover and 100% angle free of barriers. In the statistical applications, we used standardized urbanization-index values, ranging from 0 to 100. Within the figure, N represents the reference for full natural conditions; A represents a trap mostly surrounded by natural conditions, which is characterized by high forest cover and a small amount of barriers; B and C represent traps with highly urbanized surroundings. More in particular, B represents a trap surrounded by a high number of barriers and a discrete amount of forest cover, whereas C represents a trap surrounded by a low forest cover and a low presence of barriers.

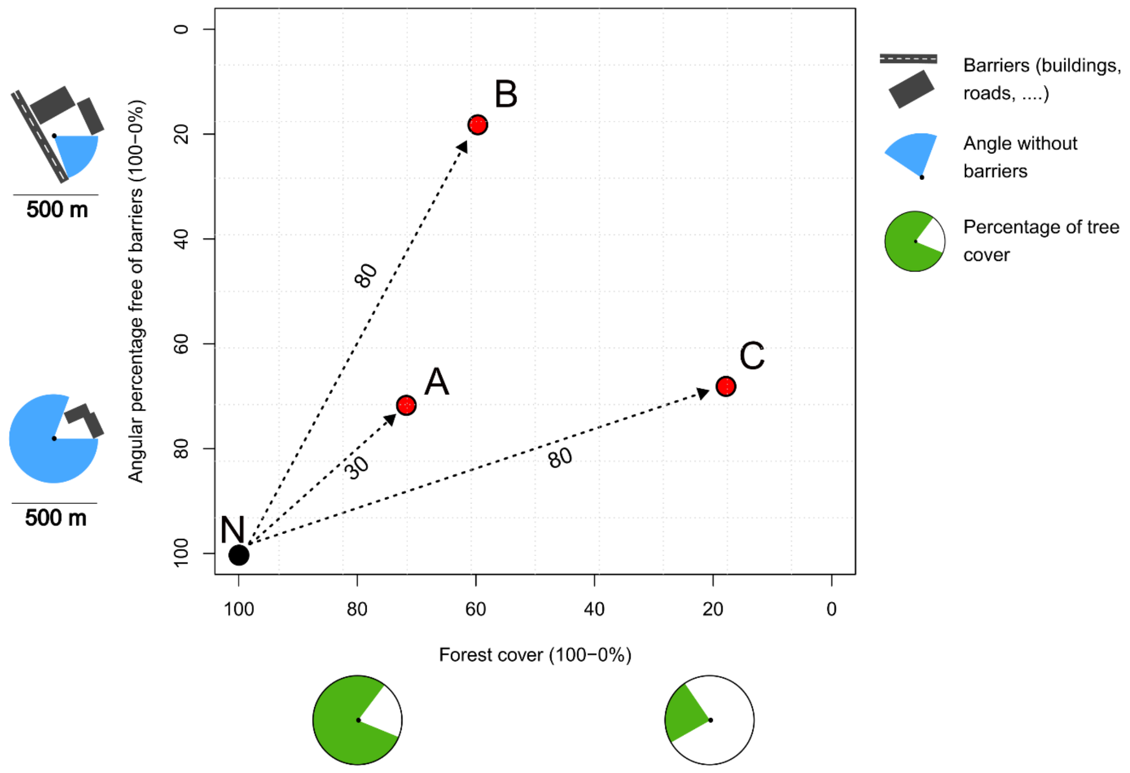

Figure S2. Relationship between sample coverage and the observed species richness at the site scale. The dashed line and grey area represent linear regression and confidence intervals, respectively. Regression is not significant (LM, $F_{1,11} = 3.84$, $p = 0.086$).

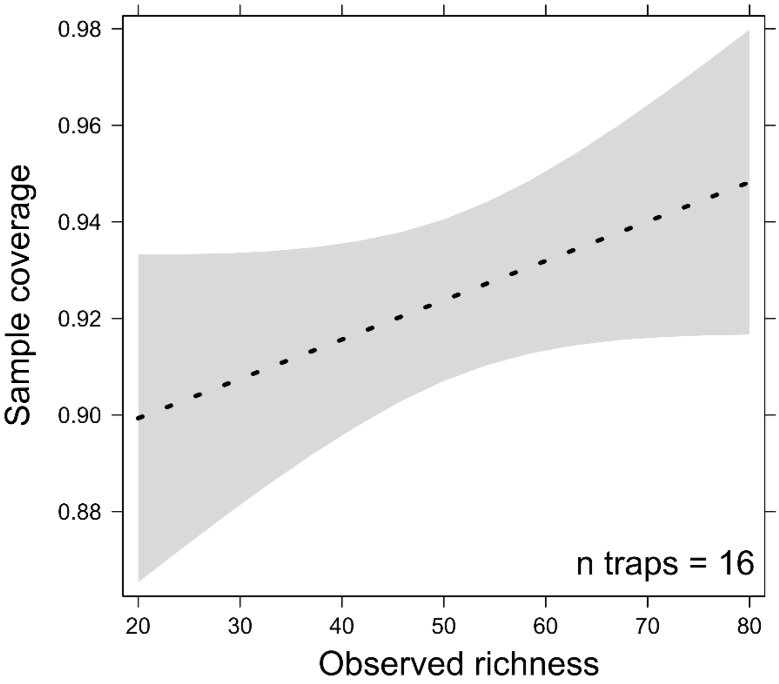

Figure S3. Average percentage of species loss depending on the reduced sampling effort under the random trap removal scenario (100 permutations).

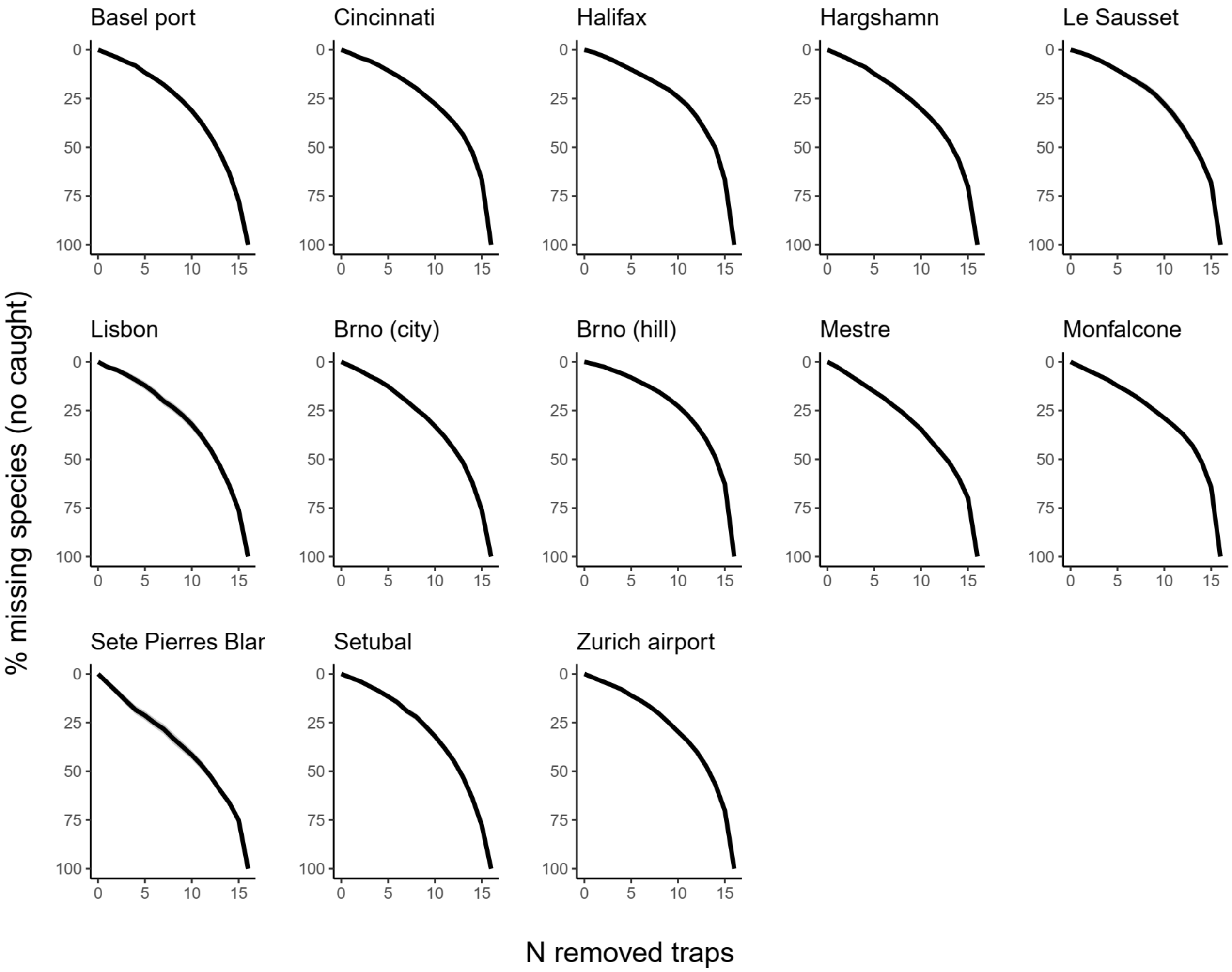

Figure S4. Average percentage of species loss depending on the reduced sampling effort under a sequential trap removal scenario based on the urbanization gradient (in blue ascending order, in red descending order).

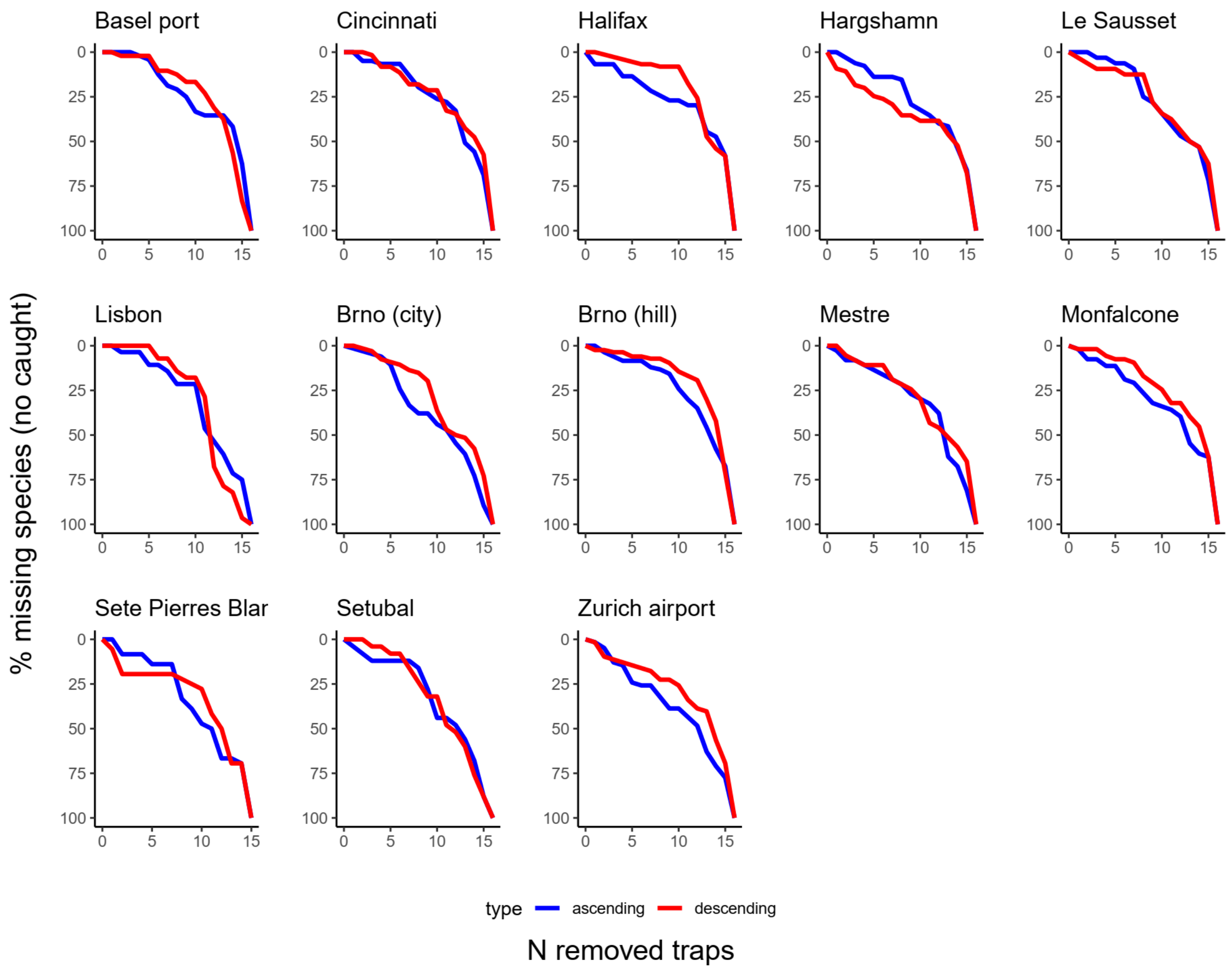

Supplement: Supplementary file 3 — Appendix S3. [file EAP-36-e70194-s003.pdf]
